# Widespread pustular eruption on paclitaxel infusion

**DOI:** 10.1016/j.jdcr.2026.03.009

**Published:** 2026-03-14

**Authors:** Gabrielle E. Gard, Travis Vandergriff, Shifa Kanjwal, Meghan Heberton

**Affiliations:** a University of Texas at Southwestern Medical School, Dallas, Texas; b Department of Dermatology, University of Texas at Southwestern Medical Center, Dallas, Texas; c Department of Internal Medicine, University of Texas at Southwestern Medical Center, Dallas, Texas

**Keywords:** acute generalized exanthematous pustulosis, adverse cutaneous reaction, AGEP, chemotherapy adverse cutaneous reaction, paclitaxel adverse cutaneous reaction, paclitaxel drug eruption, pustular drug eruption, pustular eruption, pustulosis, taxane adverse cutaneous reaction

A 61-year-old patient with locally advanced ER+/HER2-breast cancer received an initial paclitaxel infusion. Five days later, she developed erythematous patches with an intertriginous distribution and scattered monomorphic pustules on the trunk with collarettes of desquamation, involving greater than 30% body surface area (Fig 1, Fig 2, Fig 3). A broad shave biopsy showed slight epidermal hyperplasia with spongiosis and pallor with subcorneal abundant neutrophils, and complete blood count demonstrated leukocytosis (Fig 4).

**Fig 1:**
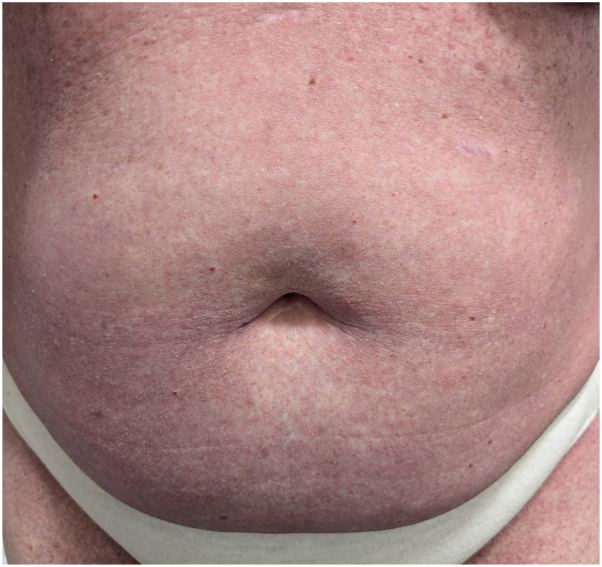
Pink patches with fine pink papules.

**Fig 2:**
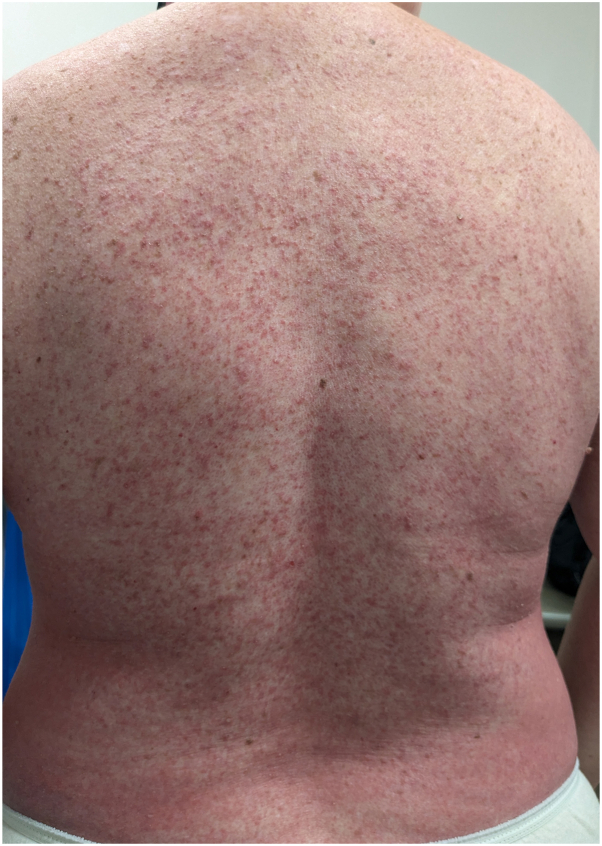
Numerous pink papules and patches.

**Fig 3:**
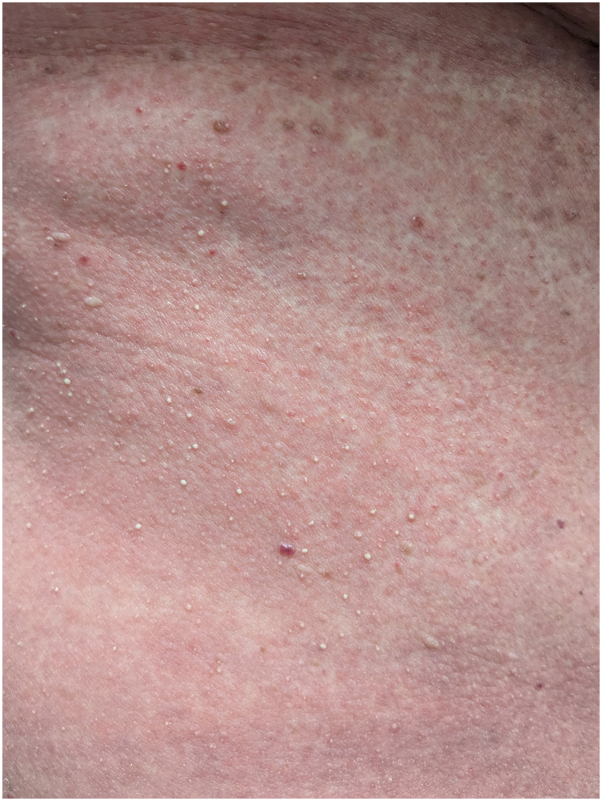
Closer evaluation demonstrates monomorphic pustules overlying pink patches.

**Fig 4:**
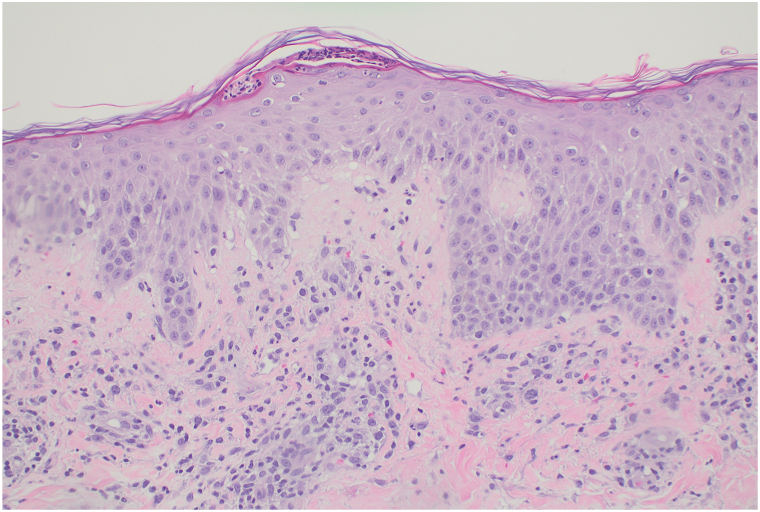
H&E stain microscopy image depicting subcorneal pustule (200× original magnification).

**Question: What is the diagnosis?**

**A.** Drug hypersensitivity syndrome (DRESS)
**B.** Toxic erythema of chemotherapy (TEC)
**C.** Pustular psoriasis
**D.** Acute generalized exanthematous pustulosis (AGEP)
**E.** Pustular vasculitis

This patient’s presentation is most consistent with AGEP associated with taxane therapy. AGEP is a rare T-cell mediated adverse skin reaction to a medication, developing 24-48 hours after administration and classically associated with antibiotics, such as sulfonamides, fluoroquinolones, and aminopenicillins.^1^ Pruritic pinhead-sized (<5 mm) sterile pustules arise in an intertriginous distribution, and on pathology, demonstrate papillary dermis edema and spongiform subcorneal pustules comprised of neutrophils and eosinophils.^2^ While AGEP is classically associated with antibiotics and other triggers, such as terbinafine, hydroxychloroquine, or diltiazem, it is important to recognize its rare occurrence with other medication classes, such as taxanes.

Taxanes are a class of cytotoxic chemotherapy agents including paclitaxel, docetaxel, and abraxane. While they are used for treating various cancers, they regularly lead to adverse dermatologic events in a dose-dependent fashion. The most common acute reactions are hypersensitivity reactions with an estimated incidence of 30% and typically occur within the first few minutes of the first or second cycle.^3^ Various rashes have been described with taxanes within the spectrum of TEC, including morbilliform reactions, intertriginous patches, and palmoplantar erythrodysesthesia. Less common reactions include drug-induced lupus erythematosus, radiation or ultraviolet recall dermatitis, and pustular eruptions (such as AGEP or folliculitis). Beyond cutaneous reactions, taxanes can also result in chronic nail changes, persistent alopecia, and mucosal changes. All taxanes can result in adverse cutaneous reactions, but paclitaxel has been reported to have a lower incidence.^3^

While this patient was ultimately diagnosed with AGEP, differential diagnoses included pustular psoriasis, DRESS, and TEC. Pustular psoriasis more commonly presents in patients with psoriasis, who often have chronic psoriatic skin or nail changes. Pustular DRESS was considered but was less likely given the drug class, shorter latency, and lack of associated systemic findings (eosinophilia, lymphadenopathy, elevated AST/ALT, or facial plethora).^1^ TEC can closely resemble AGEP as both can present in intertriginous regions, but close physical exam revealed fine monomorphic pustules, which suggested AGEP, as opposed to red patches, plaques, or bullae seen in TEC.^4^ Based on the Common Terminology Criteria for Adverse Events, this case was a grade 3 reaction.^5^ Because of the body surface area and the patient’s symptom burden, the patient was treated with topical and oral steroids, which led to resolution.^1^ After interdisciplinary discussion, it was determined that rechallenge would pose a high risk of AGEP recurrence, and the patient did not want to pursue this option. Therefore, her oncology regimen was changed to doxorubicin and cyclophosphamide.

This case highlights AGEP as a rare reaction to taxanes that should be recognized quickly for appropriate treatment and future cancer therapy counseling with the interdisciplinary team.

## Conflicts of interest

None disclosed.
